# An approach to molecular imaging of atherosclerosis, thrombosis, and vascular inflammation using microparticles of iron oxide^☆^

**DOI:** 10.1016/j.atherosclerosis.2009.10.009

**Published:** 2010-03

**Authors:** Martina A. McAteer, Asim M. Akhtar, Constantin von zur Muhlen, Robin P. Choudhury

**Affiliations:** Department of Cardiovascular Medicine, John Radcliffe Hospital, University of Oxford, Oxford OX3 9DU, United Kingdom

**Keywords:** Molecular imaging, Magnetic resonance imaging, Microparticles of iron oxide, Atherosclerosis, Thrombosis, Vascular inflammation, Ischemia-reperfusion injury

## Abstract

The rapidly evolving field of molecular imaging promises important advances in the diagnosis, characterization and pharmacological treatment of vascular disease. Magnetic resonance imaging (MRI) provides a modality that is well suited to vascular imaging as it can provide anatomical, structural and functional data on the arterial wall. Its capabilities are further enhanced by the use of a range of increasingly sophisticated contrast agents that target specific molecules, cells and biological processes. This article will discuss one such approach, using microparticles of iron oxide (MPIO).

MPIO have been shown to create highly conspicuous contrast effects on T_2_^*^-weighted MR images. We have developed a range of novel ligand-conjugated MPIO for molecular MRI of endothelial adhesion molecules, such as vascular cell adhesion molecule-1 (VCAM-1) and P-selectin expressed in vascular inflammation, as well as activated platelet thrombosis. This review discusses the application of ligand-targeted MPIO for *in vivo* molecular MRI in a diverse range of vascular disease models including acute vascular inflammation, atherosclerosis, thrombosis, ischemia-reperfusion injury and ischemic stroke. The exceptionally conspicuous contrast effects of ligand-conjugated MPIO provide a versatile and sensitive tool for quantitative vascular molecular imaging that could refine diagnosis and measure response to treatment. The potential for clinical translation of this new class of molecular contrast agent for clinical imaging of vascular syndromes is discussed.

## 1 Introduction

In clinical practice, the early identification and characterization of atherosclerotic lesions at risk of progressing to cause myocardial infarction (MI) and ischemic stroke remains challenging. X-ray arteriography is the standard clinical imaging technique used to estimate the degree of luminal stenosis, but detects only the silhouette of the vessel wall against the lumen and does not provide information on plaque composition. More sophisticated non-invasive imaging techniques are required that can accelerate and refine diagnosis, guide intervention and monitor response to therapies that may stabilize lesions and prevent future atherothrombotic events [1].

**Fig. 1:**
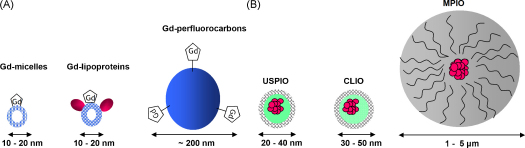
MRI relies on the delivery of relatively high payloads of either (A) gadolinium chelates or (B) iron oxide. Gd chelates decorate the surface of the carriage vehicle since Gd requires interaction with local water molecules to produce contrast effects. Iron oxide particles (size range, ∼10 nm to 5 μm) are typically contained within polymer shells. USPIO: ultrasmall particles of iron oxide; CLIO: cross-linked iron oxide nanoparticles; MPIO: microparticles of iron oxide[4].

Molecular imaging is a rapidly evolving field, which presents opportunities to shift emphasis from imaging plaque structure to reporting directly the biological processes of vascular disease at the molecular and cellular levels [2]. Specific targeting of molecules is achieved through the development of purpose-built molecular imaging probes, usually consisting of a contrast agent conjugated to specific targeting ligands [1]. Advances in nanotechnology have led to the development of an array of nano- and micrometer-sized particle contrast agents for application in molecular imaging. The most successful approaches have involved the development of molecular imaging agents that can deliver substantial payloads of paramagnetic gadolinium (Gd) chelates or superparamagnetic iron oxide to a specific molecular target (Fig. 1). Functionalization of the particle surface with reactive surface groups enables covalent conjugation of a variety of targeting ligands including antibodies, peptides, aptamers and small molecule peptidomimetics to the particle surface.

In atherosclerosis, a diverse range of molecular targets are expressed both at the endothelial surface and by cells within the vessel wall of the artery. Importantly, there is differential expression of some of these targets from early lesion formation to advanced vulnerable plaques and thrombotic complications [1,3,4]. For instance, early markers of atherogenesis include endothelial cell adhesion molecules, such as P- and E-selectin, vascular cell adhesion molecule-1 (VCAM-1) and intercellular adhesion molecule-1 (ICAM-1), which facilitate mononuclear leukocyte recruitment to activated endothelium and subsequent transmigration into the subendothelial space [5–7]. Molecular imaging of endothelial adhesion molecules has been at the forefront of this field, since the upregulation of these molecules is an early event in a broad range of vascular diseases including atherosclerosis, ischemia-reperfusion injury, ischemic stroke and cancer. Furthermore, their endothelial location makes them accessible to blood-borne contrast agents. However, one of the challenges for molecular imaging of endovascular targets is to deliver targeted contrast agents in sufficient density to detect molecular expression of relatively low-abundance targets confined to a two-dimensional endothelial monolayer and to achieve this under high physiological shear stresses.

Shapiro et al. showed that microparticles of iron oxide (MPIO) provide excellent contrast effects [8]. In this paper, we will focus on the application of ligand-targeted MPIO for molecular MRI of endothelial adhesion molecules in experimental models of acute vascular inflammation [9], ischemia-reperfusion injury [10], atherosclerosis [11] and ischemic stroke [12]. We also highlight a similar ligand-targeted MPIO strategy for MRI detection of activated platelet thrombosis. Finally, we discuss the scope for application of ligand-targeted MPIO in clinical imaging of vascular syndromes.

## 2 Molecular imaging modalities

A range of imaging techniques are currently used to image atherosclerosis including intravascular ultrasound (IVUS), multiple-row detector computed tomography (MDCT), positron emission tomography (PET), MRI and optimal coherence tomography (OCT) [13]. Of these only nuclear techniques have proven record in clinical molecular imaging. PET can assess atherosclerotic inflammatory plaque activity using exogenously administered ^18^Fluorine preparations, such as 2-[^18^F]fluoro-2-deoxy-d-glucose (FDG). This glucose analogue accumulates in metabolically active tissue, yielding a signal that is proportional to glycolytic activity. It has been shown that inflamed, metabolically active, plaques may be detectable by ^18^FDG-PET. Rudd et al. demonstrated the ability of ^18^FDG-PET imaging to highlight inflammatory activity in unstable carotid plaques in patients undergoing carotid endarterectomy, relative to the contralateral asymptomatic artery [14]. ^18^FDG was taken up by atherosclerotic plaque and selectively accumulated in macrophage-rich areas. Indeed, the first investigations of treatment effects of on plaque activity are beginning to emerge [15]. The advantages of PET are related to its extreme sensitivity and quantitative outputs. Opportunities to apply PET in the evaluation of atherosclerosis and its treatment should extend beyond macrophage imaging through the development of novel ‘radio-ligands’. For example, the development of imaging probes that target matrix metalloproteinases [16], serine proteases [17] and apoptosis [18–20], could allow the visualization of response to treatment in ‘high risk’ plaques. While PET has much higher detection sensitivity (picomolar range) than MRI, and much better tissue penetration than ultrasound or OCT, the disadvantages of PET are its limited spatial resolution, logistics of isotope provision, substantial radiation exposure and expense.

MRI has emerged as a leading non-invasive imaging modality for assessing vascular pathologies, due to its excellent spatial resolution (sub-millimeter) and soft tissue contrast and high signal to noise ratio. Using multi-contrast sequences, MRI can differentiate atherosclerotic plaque composition, based on differences in biophysical and biochemical properties such as chemical composition and concentration, water content, physical state, molecular motion or diffusion [1]. High resolution MRI can also accurately quantify plaque components such as fibrous cap thickness and size of the lipid core [21,22]. Since MRI does not involve ionizing radiation, serial imaging can be performed safely over time within the same patient. Furthermore, intravenous Gd-based or iron oxide-based MRI contrast agents can be used to improve image sensitivity to enhance differences between healthy and diseased vessels.

## 3 Gadolinium based contrast agents

Gd chelates, such as gadolinium diethylene-triamine-penta-acetic acid (Gd-DTPA), provide positive signal enhancement on T_1_ weighted MR images due to their effects on shortening water proton T_1_ relaxation times. However, Gd chelates have inherently low sensitivity (micromolar range). For molecular imaging, a number of strategies have been employed to amplify the Gd contrast effects and to deliver sufficient quantities of Gd *in vivo* in order to detect biological activity of lesions. A range of nanoparticles carrying substantial payloads of amphipathic Gd chelates embedded in their outer membrane have been constructed, including liposomes [23], perfluorocarbon lipid emulsions [24] and micelles [25,26]. Lipoprotein micelles enriched with hydrophobic Gd chelates have also been developed for the detection of macrophages within atherosclerotic plaques [27–29]. However, the relaxivity effects achievable are relatively modest, compared to superparamagnetic particles of iron oxide (SPIO) [27,30]. A further potential disadvantage of hydrophobic Gd chelates is the recently observed severe long-term toxicity effects (including nephrogenic sclerosing fibrosis: NSF) in patients with impaired renal function [31]. However, it may be the case that targeted contrast agents could be used at lower total Gd-dose, reducing the potential for this type of toxicity.

## 4 Nano- and micrometer-sized particles of iron oxide

Superparamagnetic iron oxide-based agents consist of a core of iron oxides, surrounded by a dextran or polymer coat. Iron oxide agents, shorten T_2_ and T_2_^*^ relaxation times, creating hypointense areas that appear black on the MR image on T_2_- and T_2_^*^-weighted MR images. Iron oxide agents include ultrasmall superparamagnetic particles of iron oxide (USPIO) (20–50 nm diameter), superparamagnetic particles of iron oxide (SPIO) (60 to approximately 250 nm) and micrometer-sized particles of iron oxide (MPIO) (0.9–8 μm). Iron oxide agents have superior sensitivity in MR contrast, compared to Gd [32,33]. In particular, MPIO convey a payload of iron oxide (typically 0.1–1.6 pg iron/MPIO particle), which is orders of magnitude greater than that contained in nanometer-sized particles [8]. The effects of MPIO on local magnetic field homogeneity and detectable contrast extend a distance up to 50 times the physical diameter of the microparticle, known as a contrast “blooming effect” [34]. MPIO have been shown to be useful for *cellular* MRI, enabling *in vivo* detection of single cells [35] and cell tracking [8] using only a small number of MPIO.

For *molecular* imaging of endovascular targets, MPIO offer a number of important attributes. First, the relatively large size and incompressible nature of MPIO, makes them less susceptible to non-specific vascular egress or uptake by endothelial cells than nanometer-sized particles [36], thus they can retain specificity for endothelial molecular targets. Secondly, unbound MPIO have been shown to clear rapidly from the blood (blood half-life <2 min in rats), thus minimising background blood phase contrast [37]. Conversely, USPIO have a long blood half-life (up to 24 h), which for MRI of endothelial molecular targets, may cause high background contrast for an extended period, making it difficult to distinguish specific contrast effects from normal tissue heterogeneity and other susceptibility artefacts. However, “positive” contrast MRI sequences are being developed to generate MR signal enhancement from regions containing iron oxide particles [38,39]. Thirdly, due to the contrast “blooming effect” of MPIO, a small number of MPIO can create potent hypointense contrast effects on T_2_^*^-weighted images, thereby greatly enhancing sensitivity, especially for low-abundance endothelial molecular targets. MPIO contrast effects may be readily distinguished on T_2_^*^-weighted images using *in vivo* gradient-echo MRI. MPIO have been demonstrated to provide a platform for quantitative molecular imaging of vascular endothelial targets, whereby the extent of contrast effects may directly report specific molecular endothelial expression [9–11].

Recently, SPIO with optimised high relaxation properties have been developed for quantitative *in vivo* MRI of lipoprotein metabolism [40] and ultra-sensitive detection of bacteria [41]. Cormode et al. have demonstrated that iron oxide nanocrystals can be used instead of Gd to label high density lipoprotein (HDL) particles for molecular imaging of macrophage expression in atherosclerosis [42]. Whilst effective for the applications reported, the small size of these particles is likely to be limiting to their application in molecular endothelial imaging, where the delivery of sufficient contrast volume is challenging, on the ‘planar’ target that is on the surface of the blood vessel wall.

## 5 Targeted micrometer-sized particles of iron oxide

The targeting of MPIO to specific molecules may be accomplished by the conjugation of ligands to functional groups on the surface of the microparticle. MPIO are commercially available with a variety of reactive surface groups including carboxylic acid, amine and *p*-toluene sulphydryl (tosyl) groups. These functionalized MPIO offer opportunities to covalently conjugate a range of targeting ligands including monoclonal antibodies or their immunospecific fragments F(ab), aptamers or small peptides generated by phage display or small molecule screens. Phage display provides a powerful method for the production of novel antibody or peptide ligands from libraries of bacteriophage (viruses that infect bacterial cells) using standard recombinant DNA technology. Functionalized MPIO therefore provide a versatile platform that can be readily adapted for molecular imaging of a variety of endovascular molecular targets in experimental, pre-clinical investigations of vascular inflammatory diseases. In Table 1, applications of targeted and non-targeted MPIO for molecular and cellular imaging of vascular syndromes are listed.

We have applied tosyl-activated MPIO for direct covalent conjugation of monoclonal antibodies directed against vascular endothelial adhesion molecules, including P-selectin and VCAM-1, which are involved in leukocyte recruitment during early vascular inflammation. Below, we review the application of tosyl-activated MPIO for *in vivo* molecular MRI detection of vascular endothelial adhesion molecule upregulation in diverse models of clinically important vascular pathologies including acute vascular inflammation [9], atherosclerosis [11], ischemia-reperfusion injury [10] and ischemic stroke [12]. We also highlight the application of similar cobalt-functionalized MPIO for direct covalent conjugation to histidine (His) tagged single-chain antibodies, targeted towards activated platelets in mouse models of atherothrombosis [43,44]. Finally, we discuss the potential clinical translation of ligand-targeted MPIO as a versatile adjunct in the clinical imaging arena.

## 6 Molecular imaging of acute vascular inflammation

Acute inflammation of the central nervous system (CNS) is associated with enhanced expression of endothelial adhesion molecules. Endothelial VCAM-1 and its ligand, α_4_β_1_ integrin (also called very late antigen-4, VLA-4) are key mediators of leukocyte recruitment and lesion development [45]. VCAM-1 is not constitutively expressed on the vascular endothelium but is up-regulated upon endothelial activation [46]. Furthermore, selective VCAM-1 inhibitors such as natalizumab that bind to the α_4_ subunit of α_4_β_1_ integrin are effective anti-inflammatory agents, for instance in the treatment of multiple sclerosis [47]. For these reasons, VCAM-1 is an attractive molecular imaging target of acute vascular inflammation.

**Fig. 2:**
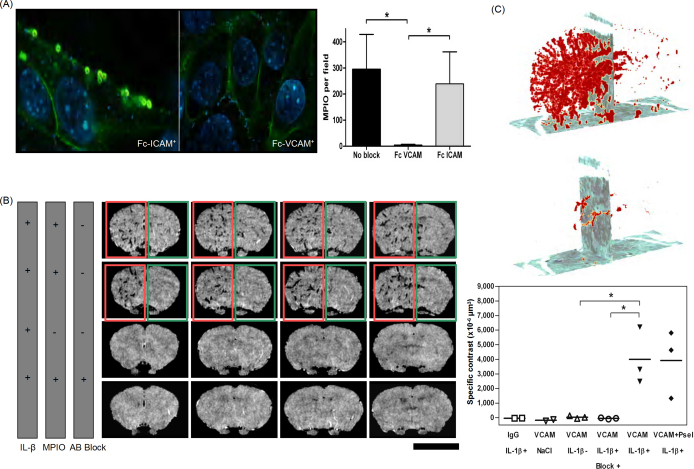
Imaging of VCAM-1 in acute inflammation. (A) Confocal microscopy of TNF-α stimulated sEND-1 cells. Green fluorescence reflects VCAM-1 expression on the cell surface. Prior incubation of VCAM-MPIO with Fc-ICAM-1 had no effect on VCAM-MPIO binding (autofluorescent green spheres), whereas pre-incubation with Fc-VCAM-1 abolished VCAM-MPIO retention, despite demonstrable VCAM-1 surface expression. Graph depicts retained VCAM-MPIO (mean ± S.D.) with and without pre-incubation with soluble Fc-VCAM-1 or Fc-ICAM-1 (^*^P < 0.0001). (B) *In vivo* T_2_^*^-weighted MR coronal images (4 images per brain; resolution ∼90 μm^3^). Intense low signal areas (highlighted with red box) on the left side of the brain reflect specific MPIO retention (VCAM-MPIO (row 1) VCAM + P-selectin MPIO (row 2)) on acutely activated vascular endothelium with almost absent contrast effect in the contralateral hemisphere (green box). No contrast effects were observed with IgG-MPIO control (row 3) or pre-treatment with VCAM-1 antibody prior to VCAM-MPIO administration, which effectively blocked VCAM-MPIO binding (row 4). Scale bar, 5 mm. (C) Three-dimensional volumetric maps of VCAM-MPIO contrast effects (red) delineate the architecture of cerebral vasculature in the IL-1β-stimulated hemisphere (left half of top image) with almost total absence of binding on the contralateral, non-activated side. The midlines are indicated by vertical sections. Pre-administration of VCAM-1 antibody abolished VCAM-MPIO retention (lower image). Quantitative analyses of MPIO contrast effects found that specific VCAM-MPIO contrast was increased >100-fold, compared with brains without IL-1β injection. Dual-targeted VCAM + P-selectin MPIO also bound specifically but did not further enhance contrast effects. Substitution of IgG-MPIO (IgG/IL-1β^+^), sham intracerebral injection (VCAM/NaCl), no intracerebral injection (VCAM/IL-1β^−^) and pre-blocking (VCAM/IL-1β^+^ with block) were not associated with specific contrast effects. Bars indicate mean values for each group (^*^P = 0.02) [9].

VCAM-1 targeted MPIO can conspicuously show upregulation of VCAM-1 in a mouse model of early cerebral inflammation by molecular *in vivo* MRI, at a time when pathology is otherwise undetectable by conventional imaging techniques [9]. MPIO (1 μm diameter), with reactive tosyl groups, were covalently conjugated to mouse monoclonal antibodies against VCAM-1 (VCAM-MPIO) or IgG isotype negative control. The capacity of VCAM-MPIO constructs for specific and quantitative binding was tested *in vitro* using a mouse endothelial cell line (sEND-1), stimulated with graded doses of tumor necrosis factor-α (TNF-α). Differential interference confocal microscopy showed a TNF-α dose-dependent increase in VCAM-MPIO binding, which co-localized with VCAM-1 immunofluorescence on the endothelial cell surface. Furthermore, VCAM-MPIO binding to stimulated cells was inhibited when the VCAM-MPIO were pre-incubated with soluble decoy VCAM-1 (mouse recombinant Fc-VCAM-1) (Fig. 2A).

For *in vivo* experiments, pro-inflammatory interleukin 1β (IL-1β) was stereotactically injected into the left corpus striatum of NMRI mice to induce acute vascular inflammation. The contralateral hemisphere received no injection and served as an internal control. VCAM-MPIO or negative control IgG-MPIO (∼4.5 mg/kg body weight) were intravenously injected 3 h after IL-1β injection and allowed to circulate for 1.5–2 h prior to MRI. To block VCAM-1 binding sites, a further group of mice were pre-treated with VCAM-1 antibody 3 h after IL-1β injection and VCAM-MPIO administered 15 min later. *In vivo* MRI was performed at 7 T using a T_2_^*^-weighted 3D gradient-echo sequence (acquisition ∼1 h; isotropic resolution 88 μm^3^). VCAM-MPIO produced highly specific hypointense signal areas in the IL-1β activated hemisphere, which delineated the architecture of activated cerebral blood vessels, with minimal contrast effects in the contralateral, unstimulated hemisphere (Fig. 2B and C). Mice that received negative control IgG-MPIO and mice pre-treated with VCAM-1 antibody prior to VCAM-MPIO injection also showed minimal contrast effects. The specificity and potency of VCAM-MPIO contrast effects were derived from a combination of targeted delivery of MPIO containing a large amount of iron oxide to sites of early inflammation and rapid clearance of MPIO from the blood which minimizes background signal. Previously, Gd-based nanoparticles conjugated to Sialyl Lewis^x^ (sLe^X^) mimetic moiety (Gd-DTPA-sLe^x^A) have been reported to detect early endothelial activation of E-selectin in a rat model of brain inflammation [48]. Detection of ICAM-1 upregulation has also been reported by *ex vivo* MRI (9.4 T) using antibody-conjugated paramagnetic liposomes [49].

## 7 Molecular imaging of atherosclerosis using dual-targeted MPIO

In the application described above, MPIO accumulate in cerebral venules, where shear stresses and flow rates are relatively low. Atherosclerosis, however, is characterized by the accumulation of lipid-rich, fibrous and cellular elements within the wall of large and medium-sized arteries, including the coronary and carotid arteries, as well as the aorta and peripheral vessels [50–52]. Molecular imaging of the vascular endothelium of large arteries presents challenges since the contrast agent has to bind in sufficient density to a two-dimensional monolayer exposed to high physiological shear stress conditions. The dynamics of leukocyte binding to activated endothelium are complex and rely on multiple receptor-ligand interactions. Initial leukocyte rolling is mediated by E- and P-selectin whereas firm adhesion to the vascular wall is mediated via integrin binding with intercellular adhesion molecule-1 (ICAM-1) and VCAM-1, with the latter more important in initiation of atherosclerosis [6,53]. VCAM-1 is not constitutively expressed but is upregulated at atherosclerosis-prone sites even before macroscopic disease is apparent, with persistent expression in more advanced atherosclerotic lesions [54,55]. Computed models of adhesion molecule dynamics predict synergistic roles for selectins and integrins with transition between rolling and firm adhesion dependent on the binding affinities and relative concentrations of receptor-ligand interactions [56,57].

**Fig. 3:**
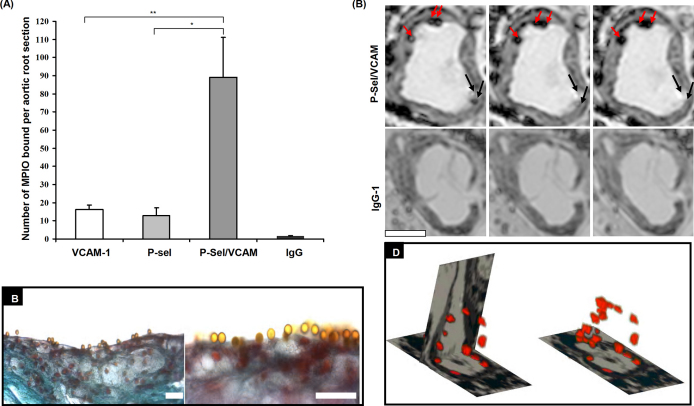
Dual-targeted MPIO binding in mouse atherosclerosis. (A) Dual-ligand MPIO recognizing VCAM-1 and P-selectin showed 7-fold enhanced binding to aortic root plaque endothelium compared to single-ligand MPIO targeting either VCAM-1 or P-selectin, following left ventricular injection. **P < 0.01; *P < 0.05. (B) Dense dual-targeted MPIO binding to endothelium overlying atherosclerotic plaque. Scale bar, 20 μm. (C) *Ex vivo* MRI of aortic roots 30 min after i.v. injection of MPIO. Dual-targeted MPIO binding appeared as distinct circular low signal areas adherent to endothelium overlying atherosclerotic plaque. Minimal contrast effects were observed with negative isotype IgG-MPIO. Scale bar, 500 μm. (D) 3D reconstruction of dual-targeted MPIO contrast effects through the aortic root [21].

In order to mimic the *in vivo* multi-step dynamics of leukocyte adhesion, we have constructed dual antibody-conjugated MPIO (4.5 μm diameter) using monoclonal antibodies to P-selectin and VCAM-1, in a 50:50 combination (PV-MPIO) [11]. As predicted by computed models, we have demonstrated using *in vivo* bioassays that dual-targeted MPIO markedly enhance binding to atherosclerotic plaque endothelium compared to single-ligand MPIO (7-fold increase in binding compared with P-selectin-MPIO and 6-fold increase compared with VCAM-MPIO) (Fig. 3A) [11]. The ability of dual-ligand PV-MPIO to bind to aortic root plaque endothelium *in vivo* was then investigated using apo E^−/−^ mice, fed a high fat diet for 26 weeks. Apo E^−/−^ mice were intravenously injected with dual-targeted PV-MPIO or negative control IgG-MPIO (30 mg iron per kg body weight) and allowed to circulate for 30 min. Mice were terminally anesthetized and the arterial tree perfusion fixed and embedded in an MR tube for high resolution *ex vivo* MRI (9.4 T). MPIO binding was readily distinguished on the arterial endothelium of plaque, providing excellent visualization by MRI (Fig. 3C). 3D reconstruction of segmented images demonstrated specific PV-MPIO binding localized to atherosclerotic plaque endothelium throughout the aortic root, with minimal retention of IgG-MPIO (Fig. 3D). No MPIO binding was observed in atherosclerosis-free areas of the ascending aorta.

We had purposely used relatively large MPIO (4.5 μm diameter) to target endothelial adhesion molecule expression in aortic atherosclerotic plaques because of their presumed superior contrast effects [8]. However, the dual-ligand conjugation protocol could be applied to smaller 1 μm diameter MPIO, which we now appreciate may be less buoyant in the circulation that these larger MPIO and therefore may exhibit effective contrast binding at lower doses. Similar dual-targeted microbubbles conjugated with Sialyl Lewis^x^ and ICAM-1 antibody have also been developed for molecular ultrasound imaging, although in that case, binding efficiency was increased only marginally by the combination. We speculate that this was due to stearic limitations resulting from the size mismatch of the glycoprotein and antibody ligands [58]. Several iron oxide nanoparticle agents conjugated to novel VCAM-1 internalising peptides, identified by phage display have also been reported. These peptides bind specifically to activated endothelium and are internalized by cells expressing VCAM-1, allowing progressive concentration by endothelial cells [59,60].

## 8 Molecular imaging of ischemia-reperfusion injury

Vascular inflammation is a key feature following ischemia-reperfusion injury (IRI) [61] in acute vascular syndromes such as myocardial infarction [62–64], stroke [65,66], cardiac surgery [67] and organ transplantation [68]. The upregulation of endothelial adhesion molecule expression persists after the ischemic event itself has resolved and therefore may provide a functional imprint of a prior ischemic insult [69]. We have recently shown that VCAM-MPIO (1 μm diameter) can detect VCAM-1 expression in a mouse model of unilateral renal IRI [10,70]. We also identified *in vitro* using TNF-α stimulated cells that VCAM-MPIO binding correlates with both VCAM-1 protein and mRNA levels, as determined by western blotting and RT-PCR respectively [10]. For *in vivo* studies, IRI was induced in male C57BL/6 mice by clamping the left renal pedicle for 30 min, while the contralateral pedicle was exposed but not instrumented [71]. After 16–18 h reperfusion, mice were intravenously injected with VCAM-MPIO or irrelevant control IgG-MPIO (4.5 mg iron per kg). A further group of mice were pre-treated with VCAM-1 antibody 15 min prior to VCAM-MPIO injection to block VCAM-1 binding sites. *In vivo* MRI (9.4 T) was performed for 90 min post-contrast injection using a double-gated 3D gradient-echo sequence, optimized to provide both bright blood and T_2_^*^-weighted contrast (resolution 100 μm^3^). Significant VCAM-MPIO binding was observed in both the medulla and cortex of IRI kidneys compared with irrelevant control IgG-MPIO. Pre-treatment of mice with VCAM-1 antibody prior to VCAM-MPIO administration abolished retention of VCAM-MPIO.

Recently, Hoyte et al. have demonstrated that VCAM-MPIO can detect unilateral cerebral ischemia in a mouse model of experimental stroke [12]. Barber et al. have also reported molecular MRI of transient middle cerebral artery brain ischemia using Gd-DTPA-sLe^x^A, targeting both P- and E-selectin, but identified limitations in contrast sensitivity [72]. Targeted ultrasound microbubbles, targeting P-selectin and ICAM-1 have been developed and applied to image mouse renal [73,74] and myocardial IRI [75,76]. Current clinical imaging techniques are hampered by an inability to define the extent and distribution of ischemia in acute vascular syndromes. The ability of our ligand-targeted MPIO approach to depict the volume of endothelial inflammation following IRI, in relation to the vasculature, may aid accelerated diagnosis of parenchymal ischemia and potentially, guide targeted interventions.

## 9 Molecular imaging of thrombus using MPIO targeted to activated platelets

**Fig. 4:**
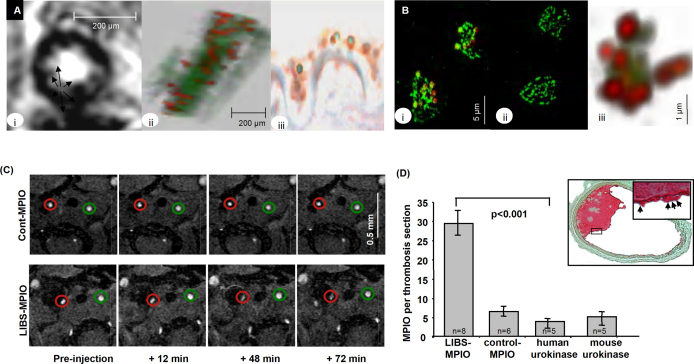
MRI of activated platelets using MPIO conjugated to single-chain antibodies directed against ligand-induced binding sites (LIBS). (A) (i) *Ex vivo* MRI of a wire-injured femoral artery exposed to LIBS-MPIO shows multiple, intensely low signal, lobulated areas at the interface between vessel wall and lumen (arrows). (ii) Three-dimensional reconstruction shows diffuse and relatively even LIBS-MPIO binding along the luminal surface of the injured femoral artery. (iii) Co-localization of LIBS-MPIO and platelets was confirmed using immunohistochemistry for CD62 [43]. (B) Confocal microscopy of human platelets immobilized on fibrinogen and detected by immunofluorescence using CD62 antibody (green) [43]. (i) LIBS-MPIO (red) show specific binding to platelets. (ii) No binding was observed with control-MPIO. (iii) 3D rendering shows multiple LIBS-MPIO binding to clusters of activated platelets, via GPIIb/IIIa. (C) *In vivo* T_2_^*^-weighted MRI after carotid artery injury [44]. Transverse sections demonstrate the injured right carotid artery (red circle), and the non-injured left carotid artery (green circle). Following LIBS-MPIO injection, there is increasing signal drop at 12, 24, and 72 min compared with preinjection and the non-injured left carotid artery. For control-MPIO, signal intensity is similar at 12, 48, and 72 min in both vessels. (D) Immunohistochemistry of wall-adherent thrombus in a LIBS-MPIO-injected animal. In the inset, arrows depict bound MPIO on the thrombus surface (thrombus area itself appears red). Quantification of MPIO bound to wall-adherent thrombosis shows significantly higher LIBS-MPIO binding compared to control-MPIO or to mice treated with human urokinase or mouse recombinant urokinase to induce thrombolysis, prior to LIBS-MPIO administration [44].

Activated platelets are known to be involved both in the initiation of atherosclerosis and in advanced atherosclerotic events, such as plaque rupture and thrombus formation [77,78,79]. The platelet-specific glycoprotein GP IIb/IIIa receptor (CD41/CD61, also known as α_IIb_β_3_ integrin) mediates the final common pathway of platelet aggregation via fibrinogen and is key to thrombus formation [80]. Recently, a single-chain antibody that specifically recognizes ligand-induced binding sites (LIBS) on GP IIb/IIIa receptors, which become exposed only upon activation by receptor-ligand binding, has been developed [81–83] The LIBS antibody has been shown to bind only to activated platelets, e.g. when adherent to damaged endothelium, and does not to bind to non-activated circulating platelets. von zur Muhlen et al. recently applied cobalt-functionalized MPIO (1 μm diameter) conjugated to the histidine tag of LIBS single-chain antibodies (LIBS-MPIO) for the detection of activated platelets in a mouse model of endovascular platelet aggregation using *ex vivo* MRI (11.7 T) (Fig. 4A) [43]. LIBS-MPIO agent have also been applied in a mouse model of wall-adherent, carotid thrombosis for the detection of platelet-rich thrombi by *in vivo* MRI (Fig. 4C) [44]. LIBS-MPIO reliably tracked a reduction in thrombus size in response to pharmacological thrombolysis treatment with urokinase (Fig. 4D). LIBS-MPIO have also been used to detect human platelet aggregates in explanted symptomatic carotid artery plaque specimens by *ex vivo* MRI (9.4 T) [44] and human platelet-rich clots *in vitro* using clinically relevant magnetic field strengths (3 T) [84].

## 10 Clinical translation of MPIO

For clinical purposes, the commercial MPIO that we have used are non-biodegradable, due to their polyurethane coat, and are not suitable for human applications. However, for clinical translation, it should be feasible to synthesize biodegradable MPIO with suitable modification of the surface coat, similar to the iron oxide containing contrast media that are already in clinical use [85]. In fact, the development of biodegradable MPIO suitable for clinical use is already underway [86–89]. Our ligand-targeted MPIO approach has focused on the use of monoclonal antibodies. Potential immunogenicity can be attenuated by the use of modified or “humanized” antibodies [90] and single-chain antibodies [43,82]. However, antibody production on a scale required for clinical application is also relatively complex and expensive. Sugar-based ligands, such as sLe^X^, have recently been applied to develop novel glyconanoparticles [91], and may prove useful ligands for clinical application.

**Fig. 5:**
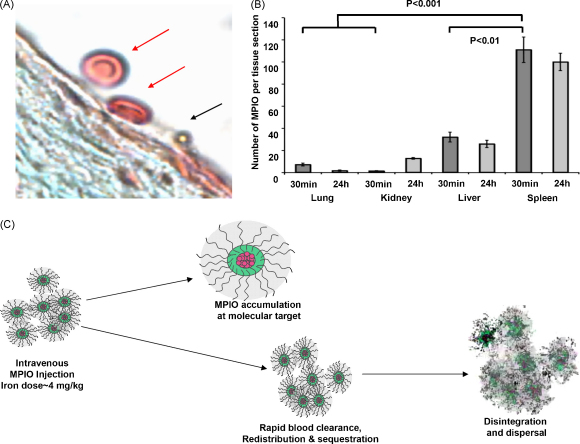
(A) Histological section depicting the considerably smaller size of MPIO (1 μm diameter) (black arrow) bound to endothelium overlying aortic root plaque, compared to adjacent red blood cells (red arrows). (B) *In vivo* biodistribution studies show that MPIO retention by the lungs is minimal, while MPIO uptake by the spleen and liver is rapid [43]. (C) Schematic representation of the biological handling properties of biodegradable MPIO. Efficient removal of contrast agent from the circulation via the reticulo-endothelial system is advantageous for *in vivo* imaging, where background blood MPIO may otherwise obscure specifically bound contrast. Disintegration and dispersal of the dextran coat and iron via normal iron handling pathways is required.

For our *in vivo* imaging studies, the dose of iron that we have used (4.5 mg iron/kg body weight) has been well tolerated, with no animals showing any short-term ill effects. This iron dose closely reflects the dose of USPIO (2.6 mg iron/kg body weight) used extensively for human oncological MRI [92]. Further dose-ranging studies will determine whether the iron dose can be reduced further. Importantly, MPIO, which are considerably smaller than red blood cells (Fig. 5A), do not induce any evidence of hemorrhage or tissue infarction due to small vessel ‘plugging’ [11] and are rapidly sequestered by the liver and spleen (Fig. 5B) [43]. Another important advantage of the MPIO approach is the ability to relate specific MPIO binding to the blood vessel architecture [10]. The anatomical distribution together with the degree and timing of contrast binding may add further functional specificity for vascular pathological processes under evaluation.

## 11 Conclusions

In summary, we have developed a new approach to molecular imaging of endovascular targets using ligand-conjugated MPIO, which we have applied in a diverse range of vascular syndromes including acute brain inflammation, atherosclerosis, renal ischemia-reperfusion injury, ischemic stroke and thrombus formation. The versatility of functionalized MPIO and the potency of the contrast effects in detecting relatively low-abundance endothelial molecular targets, offers a potentially valuable platform for accelerating diagnosis and guiding specific treatment of vascular inflammatory diseases.

## Figures and Tables

**Table 1:** Approaches to molecular and cellular imaging of vascular syndromes using microparticles of iron oxide.

Biological process	Target	MPIO agent	MR field strength (T)	Disease model
*Targeted*				
Inflammation	VCAM-1	VCAM-MPIO (1 μm)	7 T *in vivo*	Acute inflammation [9]
			7 T *in vivo*	EAE [88,89]
			9.4 T *in vivo*	IRI [10]
			7 T *in vivo*	Experimental stroke [12]
	P-selectin + VCAM-1	P/V-MPIO (4.5 μm)	11.7 T *ex vivo*	Atherosclerosis [11]
Activated platelets	GP IIb/IIIa	LIBS-MPIO (1 μm)	9.4 T *ex vivo*	Atherothrombosis [37]
			7 T *in vivo*	Infection [90]
			1.5 and 3 T *ex vivo*	Human platelets [38]
			9.4 T *in vivo*	Thrombosis [38]

*Untargeted*				
Macrophage tracking	Macrophages	MPIO (0.9 μm)	4.7 T *in vivo*	Cardiac allograft rejection [36]
			4.7 T *in vivo*	Heart transplant rejection [91]
Stem cell migration	Stem cells		1.5 T *in vivo*	Myocardial infarction [92]

MPIO: microparticles of iron oxide; VCAM-1: vascular cell adhesion molecule-1; IRI: ischemia-reperfusion injury; EAE: experimental allergic encephalomyelitis; LIBS: ligand-induced binding sites; P/V-MPIO: P-selectin and VCAM-1 antibody-conjugated MPIO.
